# Functional Properties of Antimicrobial Neem Leaves Extract Based Macroalgae Biofilms for Potential Use as Active Dry Packaging Applications

**DOI:** 10.3390/polym13101664

**Published:** 2021-05-20

**Authors:** A. A. Oyekanmi, U. Seeta Uthaya Kumar, Abdul Khalil H. P. S., N. G. Olaiya, A. A. Amirul, A. A. Rahman, Arif Nuryawan, C. K. Abdullah, Samsul Rizal

**Affiliations:** 1 School of Industrial Technology, Universiti Sains Malaysia, Penang 11800, Malaysia; abdulkan2000@yahoo.com (A.A.O.); ck_abdullah@usm.my (C.K.A.); 2 Department of Industrial and Production Engineering, Federal University of Technology, PMB 704 Akure, Nigeria; ngolaiya@futa.edu.ng; 3 School of Biological Sciences, Universiti Sains Malaysia, Penang 11800, Malaysia; amirul@usm.my; 4 School of Physics, Universiti Sains Malaysia, Penang 11800, Malaysia; arazhar@usm.my; 5 Department of Forest Products Technology, Faculty of Forestry, Universitas Sumatera Utara, Medan 20155, Indonesia; arif5@usu.ac.id; 6 Department of Mechanical Engineering, Universitas Syiah Kuala, Banda Aceh 23111, Indonesia; samsul_r@yahoo.com

**Keywords:** *Azadirachta indica*, seaweed, characterization, antimicrobial, biodegradable, composite, packaging

## Abstract

Antimicrobial irradiated seaweed–neem biocomposite films were synthesized in this study. The storage functional properties of the films were investigated. Characterization of the prepared films was conducted using SEM, FT-IR, contact angle, and antimicrobial test. The macroscopic and microscopic including the analysis of the functional group and the gas chromatography-mass spectrometry test revealed the main active constituents present in the neem extract, which was used an essential component of the fabricated films. Neem leaves’ extracts with 5% *w*/*w* concentration were incorporated into the matrix of seaweed biopolymer and the seaweed–neem bio-composite film were irradiated with different dosages of gamma radiation (0.5, 1, 1.5, and 2 kGy). The tensile, thermal, and the antimicrobial properties of the films were studied. The results revealed that the irradiated films exhibited improved functional properties compared to the control film at 1.5 kGy radiation dosage. The tensile strength, tensile modulus, and toughness exhibited by the films increased, while the elongation of the irradiated bio-composite film decreased compared to the control film. The morphology of the irradiated films demonstrated a smoother surface compared to the control and provided surface intermolecular interaction of the neem–seaweed matrix. The film indicated an optimum storage stability under ambient conditions and demonstrated no significant changes in the visual appearance. However, an increase in the moisture content was exhibited by the film, and the hydrophobic properties was retained until nine months of the storage period. The study of the films antimicrobial activities against *Staphylococcus aureus* (SA), and *Bacillus subtilis* (BS) indicated improved resistance to bacterial activities after the incorporation of neem leaves extract and gamma irradiation. The fabricated irradiated seaweed–neem bio-composite film could be used as an excellent sustainable packaging material due to its effective storage stability.

## 1. Introduction

The Synthetic petroleum-based polymers have been widely utilized as packaging materials due to their relative abundance including their good mechanical and thermal properties [1]. Over the years, the use of synthetic and other non-biodegradable polymeric packaging materials have generated a lot of ecological concerns as a result of their persistence in the environment after use [2]. In recent years, environmental awareness have increased towards the utilization of eco-friendly, renewable, and biodegradable biopolymers for sustainable packaging applications [3]. Biopolymers such as protein [4], chitosan [5], and starch [6] have been considered for packaging due to their degradability potentials and its ability to enhance biodegradability when mixed with other biodegradable materials [7].

However, the challenge of poor mechanical properties and high water barrier permeability is a major drawback that limits their application for packaging. Seaweed, which is a natural macromolecular biopolymer, has the capacity to be developed as films [8]. Its intrinsic qualities such as biodegradability, good oxygen barrier, and non-toxic behavior have been considered as a suitable functional material for edible packaging applications [9]. Seaweed can be effectively applied as packaging material because it is potentially enriched in carbohydrate which can be utilized as a backbone of biopolymer films [10]. These properties have attracted remarkable interests in seaweed biopolymers for the formation of biofilms even though its inherent relatively poor mechanical and water barrier behavior limits its wide application for packaging [11]. Researchers have reported that some of these challenges that limit the application of seaweed biopolymer for packaging can be overcome by the incorporation of cellulose nanocrystals (CNCs) into the polymer matrix to improve the mechanical and barrier properties [12]. Studies have been reported using micro crystalline cellulose (MCC) as a reinforcement agent for improved packaging [13]. 

The isolation of MCC from bamboo precursor has been utilized as reinforcement material in seaweed composite films [14]. The use of ionizing rays such as ultraviolet (UV) irradiation [15]. Gamma irradiation (γ-irradiation) have also been reported for the stability and enhancement of the properties of polysaccharides based biofilms [16]. Furthermore, the use of plant extracts as additives known to contain phytochemical substances has the potentials to improve the antimicrobial properties by hindering the growth of pathogens in biofilms. Plant extracts from olive leaves [17] and basil leaves [18] have been reported for the improvement of seaweed biofilms. In some cases, seaweed extracts were applied to improve the biological activities of biofilms. Kadam et al. [19] have reported the use of *Ascophyllum nodosum* for the improvement of the physico-chemical and antioxidant properties of gelatin and sodium caseinate biofilms. Neem leaves have provided improved antimicrobial activities in biofilms as suitable additives to enhance the properties in order to ensure food safety towards sustainable packaging applications. Neem leave extracts have received a remarkable amount of interest due to their composition of phenolic compounds that enables strong antimicrobial activities; they also exhibit high anti-bacterial activity against Gram-positive bacteria in food borne pathogens [20]. 

The long-term stability of functional properties of films is essential during storage to ensure the quality of the stored products. Packaging under this condition has the potential to protect stored products against discoloration, deterioration, pathogenic activities, and textural changes. However, a sustainable approach to maintain the stability and functional properties of film packaging bio-materials is a major concern during storage [21]. The incorporation of extracts into the film structure could improve the physical and chemical properties of the film [22]. Furthermore, physico-chemical changes may occur in the films as a result of factors such as storage temperature, storage period, and relative humidity (RH) [23]. Similarly, the tensile properties of the film, such as the tensile strength, and elongation at break including the water barrier properties of the films could be enhanced by the addition of plant extracts into the matrix [17]. In view of the significance of the ageing challenge, no study has reported the potentials of the functional storage properties of gamma irradiated seaweed–neem films for improved packaging applications. Therefore, this current study aims to evaluate the suitability and application of seaweed-based neem biofilm as a sustainable packaging material. Gamma irradiated seaweed–neem films were stored for 24 months under ambient condition (25 °C; RH: 50%). The physical and chemical properties of the irradiated composite films were determined. The tensile strength, tensile modulus, and elongation at break of the films including surface toughness were investigated according to standard methods. The effects of ambient condition on the appearance, moisture content, water barrier properties, and antimicrobial activities of the irradiated films were investigated every three-month interval. 

## 2. Materials and Methods

### 2.1. Materials

The neem leaves were harvested from Bukit Mertajam, Penang. Macrolagae red seaweed was purchased from Green Leaf Synergy Sdn. Bhd Tawau Sabah, Malaysia. 95% pure ethanol of vapour density 1.59, boiling point 78.3 °C, and density of 0.789 g/mL was purchased from Sigma Aldrich (St. Louis, MO, USA). In addition, the glycerol (boiling temperature 182 °C/20 mmHg, melting point 20 °C and density of 1.25 g/mL) used in the study was supplied by Sigma-Aldrich (St. Louis, MO, USA). 

### 2.2. Extraction and Characterisation of Neam Leaf Exract

The fully matured Azadirachta indica (neem) leaves were collected and washed under running tap water to remove any dirt prior to the drying process. The leaves were air dried for 20 days, then ground into a powder and stored in airtight bottles. Ethanol was used for the isolation of the neem leaves extract from its leaves as described by [24] with slight modification. The neem leaves powder (190 g) was soaked in 95% ethanol (1900 mL) in a covered beaker and placed in a reciprocating shaker at 28 °C for 24 h. The whole extract was filtered using Whatman No. 42 (125 mm) filter paper and cotton wool. The obtained filtrate solution was concentrated and ethanol was evaporated using a vacuumed rotary evaporator (Buchi Rotavapor R-200, Flawil, Switzerland) with the hot water bath set at 40 °C to form a paste. The extracted paste was dried further in the oven at 40 °C to get a thicker paste (11.27 g). The dried extracts were kept in the refrigerator at 4 °C for further use. The percentage of crude ethanol extract yield was calculated based on the weight of dried and ground neem leaves.

The preparation of the samples for microscopic investigation of the anatomical sections were carried out according to the methods described by [25] with modifications. Morphological structural analysis of the *Azadirachta indica* leave extracts was investigated to examine the physical properties of the extracts. Prior to the investigation, the leaf tissues were immersed in 10% buffered formalin. After that, the tissues were dehydrated using different percent concentrations of alcohol (50%, 70%, 80%, 90%, 95%, and 100% alcohol) and were later cleared in xylene and embedded in paraffin wax. Lastly, 5 mm cross-sections of the blocks were mounted on slides and stained with 0.5% methylene blue and were examined under a light microscope [26].

The functional properties of the neem leaves extracts was examined using the Fourier transform infrared (FTIR) spectroscopy (Spectrum 8900 IR Spectrometer, Shimadzu, Kyoto, Japan). The powdered Azadirachta indica leaves were mixed with potassium bromide (KBr) salt, using mortar and pestle, and were compressed into a thin pellet. The leaves extract was treated for FT-IR analysis with a scan range—from 500 cm^−1^ to 4000 cm^−1^ and the infrared spectra were recorded in transmittance mode. 

Gas chromatography–mass spectrometry (GC–MS) analysis was conducted on the neem leaves crude extract using Shimadzu GC–MS-QP2010 Ultra GC–MS for the identification of the phytoconstituents. The leaves’ extracts were analyzed in paste form using the methods of [27] with slight modifications. The analysis was carried out using a Hewlett Packard 6890 series Gas Chromatograph with 5973 N Mass Selective Detector and Chemstation Data System (Agilent Technologies, Santa Clara, CA USA) equipped with a fused silica capillary column (30 mm × 0.25 mm, film thickness 0.25 μm). An electron ionization system with ionization energy 70 eV was used for the detection of the compounds. Inert gas helium was used as a carrier gas at constant flow rate of 1 mL/min. Mass transfer line and injector temperatures were set at 280 °C. The oven temperature was set with initial temperature at 50 °C for 5 min and was later held for 10 min and finally raised to 300 °C at 25 °C/min. The neem leaves paste was diluted with appropriate solvent (1/100, *v*/*v*), and then filtered. The particle-free diluted neem leaves extract (1 μL) were obtained in a syringe and injected into injector with split mode at a split ratio of 1:120. The percentage composition of the neem leaves extract constituents was expressed in terms of percentage peak area. The chemical compounds were identified and characterized in the crude extract based on GC retention time. 

### 2.3. Preparation of Seaweed-Based Film Incorporated with Neem Leaves and Gamma Irradiated Film 

About 6 g (1%, *w*/*v*) seaweed was dissolved in 300 mL distilled water and stirred continously for 15 min after which 1.8 g of glycerol was added to 50 *w*/*w* % seaweed and was homogenously stirred for 25 min. In addition, 5% *w*/*w* neem leaves extract was added to the seaweed film structure, based on our previous work on the incorporation of different neem leaves extract in the seaweed-based film [28], and was cast on a Teflon-coated plate. The casted solution mix was then dried in an oven at 40 °C for 24 h and stored in a desiccator at a relative humidity of 53% for 72 h for analysis [22]. The evaluation on the mechanical, barrier, and antimicrobial characteristics of the seaweed–neem composite film and irradiated seaweed–neem film were conducted and compared to the seaweed-based film (control film).

### 2.4. Characterization of Functional Properties of Seaweed, Seaweed–Neem, and Seaweed–Neem Gamma Irradiated Film

The functional properties of seaweed, seaweed–neem and gamma-irradiated seaweed–neem films were investigated. FT-IR analysis was conducted on the films. The samples were oven-dried at 60 °C for 24 h before analysis. The samples were analyzed using the FT-IR spectroscopy within the wavelength of 500 and 4000 cm^−1^. The infrared spectra were obtained in transmittance mode. Tensile properties of the films were investigated using Miniature Tensile Tester MT1175 (Dia-Stron Limited Instruments, Andover, UK). The tensile tester was used for the analysis using UvWin1000 software (Dia-Stron Limited Instruments, Andover, UK), and test samples were characterized according to ASTM D882-12 [29] standard. A 150 mm length by 5 mm width cross-section of the test samples were used for the investigation. The thickness of the films was obtained using a digital micrometer. The thickness of the film was evaluated as the mean of three random measurements for the segments of the films. The value of the tensile strength and elongation at break was obtained as the mean of five measurements of each test sample. The tensile fracture surface morphology of the films was investigated using the scanning electron microscopy (SEM), Zeiss EVO MA10 (Carl Zeiss, Oberkochen, Germany). The film samples were placed in the SEM holder with double-sided electrically conducting carbon adhesive tape to prevent a surface charge from forming on the samples when they were exposed to the electron beam. The films’ surfaces were then coated with a thin gold-palladium layer before investigations were carried out to enhance the conductivity of the samples using a Polaron (Fisons) SC515 sputter coater (Fison Instruments, VG Microtech, Susse, UK). The SEM applied a focused beam of high energy electrons to produce a variety of signals at the surface of the solid specimens at 7000× magnifications to obtain the cross-section of the fractured surface morphology of the films. Thermal properties of the films were examined using the thermogravimetric analysis (TGA) using (Mettler-Toledo thermogravimetric analyzer model TGA/DSC 1, Greifensee Switzerland). About 10 mg of films were weighed in an alumina crucible and were heated in a thermo gravimetric analyzer from 30 °C to 800 °C at the heating rate of 10 °C/min under flow of atmospheric nitrogen. The onset temperature (T_on_), the maximum temperature (T_max_) of decomposition and the (%) residual were determined from the temperature profile from the TGA curve. The residue was expressed as the residual after weight loss at 800 °C.

### 2.5. Characterization of Storage Properties of Optimized Seaweed–Neem Gamma Irradiated Film

The seaweed–neem gamma irradiated film with 1.5 KGy was used for this study. The film was tightly packed in a plastic container and sealed. The packaged samples were stored at temperature of 25 ± 2 °C and at a relative humidity of 50 ± 5% RH for two years. The packaging materials were analyzed to study the effect of moisture content, water barrier properties and antimicrobial analysis. The film was used as the control for determining the stability of the film at 0 day of storage. 

Moisture absorption capacity of the irradiated films were investigated at different intervals of storage period (3, 6, 9, 12, and 24 months). The percent moisture content of the films was examined at room temperature in the desiccators and at controlled RH using silica gel. The test samples were weighed under a controlled chamber at temperature and RH of 25 ± 2 °C and 50 ± 5%, respectively. The films were fully dried and weighed after which they were put in plastics and placed in the desiccator. The films were weighed again at intervals of the predetermined storage period. The percent moisture content was evaluated from the measured wet weight (*W_w_*) and dried weight (*W_d_*) as follows:

(1) %MC=Ww−WdWd

The water contact angle measurements were evaluated using a goniometer model optical contact angle (OCA) 15EC, Filderstadt, Germany. The irradiated films were cut to 1 cm × 1 cm dimension and properly placed on a flat edge that is attached to the contact angle analyser. In addition, 3 µL droplets of deionized water were placed on the surface of the film. 

Average contact angle from measurements taken from 10 different positions of the film were recorded. The test samples were conditioned at 50% RH before measurements were taken. The color analysis of the irradiated film during the storage period was conducted using a colorimeter spectrophotometer, Hunter Associates Laboratory, Inc., Reston, VA, USA. The values of ∆E for the analysis of color changes exhibited by the irradiated film were evaluated according to [12]. The values were evaluated as a function of the reference standard from the calibrated equipment as L* = 93.42, a* = −1.14, b* = 5.01. 

Antimicrobial analysis was carried out at 37 °C for 24 h on a 6 mm disk diameter films using agar diffusion assay and pathogens *Staphylococcus aureus* and *Bacillus subtilis*. The diameter of the inhibition zone measured in millimeters was obtained using a caliper. All analyses were conducted in triplicate. The analysis was carried out on day 0 at 3-month intervals until 24 months were completed. Three replicate samples were prepared for each storage day. 

## 3. Results

### 3.1. Properties of Neam Leaf Exract

**Figure 1:**
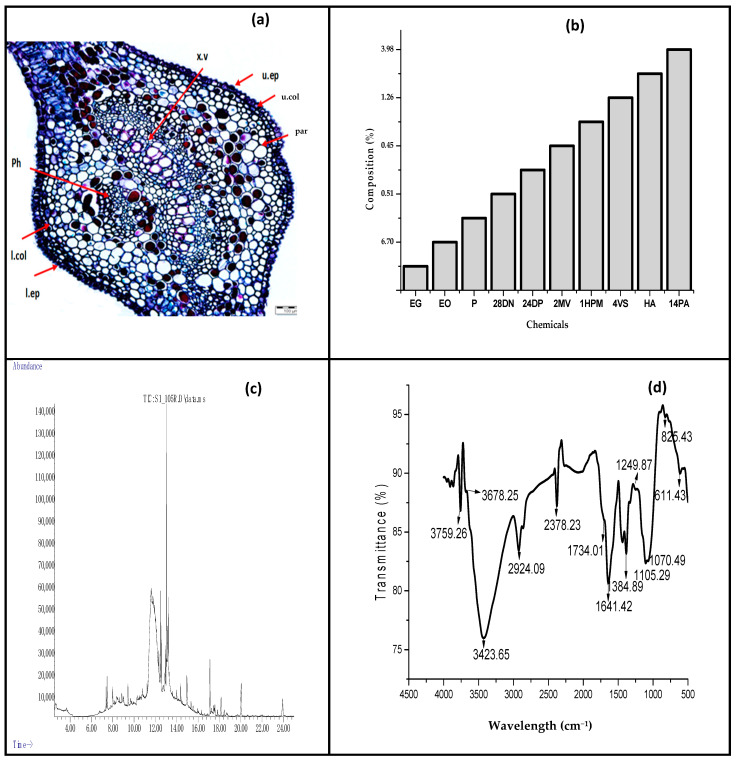
(**a**) Cross section of the leaf of *Azadirachta indica.* Magnification: 40×. col., collenchyma; ep., epidermis; x.v., xylem vessel; Ph., phloem; par., parenchyma; u.ep., upper epidermis; l.ep., lower epidermis; u. col., upper collenchymas; l. col., lower collenchymas; (**b**) chemical composition of ethanol crude neem extracts; EG represents Ethyl.alpha.-d-glucopyranoside, EO illustrates Ethyl 9, 12, 15-octadecatrienoate, P indicates Phytol. Similarly, 28DN represents 28-Deoxo-Nimbolide, 24DP represents 2,4-Dimethoxyphenol, 2Mv illustrates 2-Methoxy-4-vinylphenol and 1HPM, 4V5, HA and 14PA represent 1H-Pyrrole, 4-vinyl-syringol, Hexadecanoic acid, and 14-Pentadecenoic acid, respectively; (**c**) GC-MS analysis which identified different organic compounds from the ethanol crude extract of neem leaves; (**d**) FT-IR spectra of Neem leaves fine powder.

The surface properties of the neem leave extract used for the fabrication of film are illustrated in Figure 1a–d. The physical appearance of *Azadirachta indica* leaves indicated fresh and matured neem leaves with unequally pinnate, shiny, and dark glossy green at maturity. The average length of the leaves was within 20 to 40 cm long. The cross section of the leaflets were 3 to 8 cm long, 1.2 to 4 cm width with noticeable sickle-shaped, slightly denticulate, petiolate, and asymmetric base with a dark green color on the upper surface and lighter green color on the lower surface [30]. The petiole consists of a long and cylindrical surface with a swollen enlarged base. The average length and cross-sectional diameters were 6–9 cm and 0.1–0.3 cm, respectively. The rachis consist of long, cylindrical shape with average length of 15–20 cm and diameter of 0.15–0.3 cm. The miocrostructure of the neem leave as illustrated in Figure 1a indicated that the photomicrograph of the transverse section of *Azadirachta indica* leaf revealed the presence of the upper and lower epidermis, vascular tissues (xylem and phloem), mesophyll with palisade layer, parenchyma cells, and collenchyma cells. The upper epidermis consists of polygonal, slightly axially elongated cells with straight anticlinal walls, covered with slightly striated and thick cuticle with lower number of stomata. The structure of the lower epidermis consists of axially elongated cells with slightly wavy anticlinal walls which were covered with faintly striated cuticle and anomocytic stomata. Furthermore, the vascular bundles in the midrib region were arranged in two crescent-shaped groups forming an almost continuous ring. The observed structures of the leaf were comparable to [30].

The ethanol crude extract of neem leaves was analyzed using GC–MS and fragmentation patterns of the mass spectra were compared with those of the known compounds stored in the Chemstation Data System. The percent chemical composition of the constituents of the ethanol crude extracts is presented in Figure 1b. The result of GC-MS revealed that the identified compounds in the neem crude extracts were from sources such as hydrocarbon, terpenoids, phenolic, alkaloids, and their derivatives. These identified active compounds are considered to be plant defense systems and are utilized as natural antioxidant, antimicrobial agents. Furthermore, they are valuable compounds used in different medicinal formulations [31]. It was indicated that the crude extract was predominantly composed of Ethyl.alpha.-d-glucopyranoside (53.59%). The GC–MS data compared favorably with those reported by [32], indicating the presence of similar bioactive compounds. From the results, it was observed that the presence of 3, 7, 11, 15-tetramethyl-2-hexadecen-1-ol known as Phytol, 9, 12, 15-Octadecatrienoic acid known as Linoleic acid; α-Linolenic acid and Hexadecanoic acid known as Palmitic acid were the major components in the neem extract. Phytol is reported to have antioxidant, antiallergic, antinociceptive and anti-inflammatory activities [33]. Similarly, Palmitic acid is reported to possess cholesterolaemic and antibacterial effects [34]. The major chemical constituents identified in the neem extracts are illustrated in Figure 1c. 

The FT-IR spectra of the neem leaves powder are shown in Figure 1d. The peak found at 3423 cm^−1^ is attributed to stretching vibration of the O–H functional group for an intramolecular hydrogen bond or a phenolic group of neem leaves. The strong stretching band is also attributed to the presence of N–H stretching and bending vibration of amine group NH_2_. The absorption peak at 2924 cm^−1^ corresponds to C–H stretching vibration modes in the hydrocarbon chains, while the band corresponding to the stretching vibrations of the carbonyl functional group in ketones, aldehydes, and carboxylic acids were assigned at 1734 cm^−1^ [35]. The absorption at 1641 cm^−1^ is attributed to the amide C=O stretching indicating the presence of –COOH group in the neem leaves and the peak at 1384 cm^−1^ corresponds to stretching vibrations that originated from the C–H stretching, CH_2_ wagging, C=O, and C–O bonds of acetyl esters, respectively. The positions of the aliphatic C–C were found at 1249 cm^–1^, and the band at 1070 cm^−1^ in powdered neem leaves was assigned to C–O stretching vibration.

### 3.2. Functional Properties of Seaweed, Seaweed–Neem, Seaweed–Neem Gamma Irradiated Film

**Figure 2:**
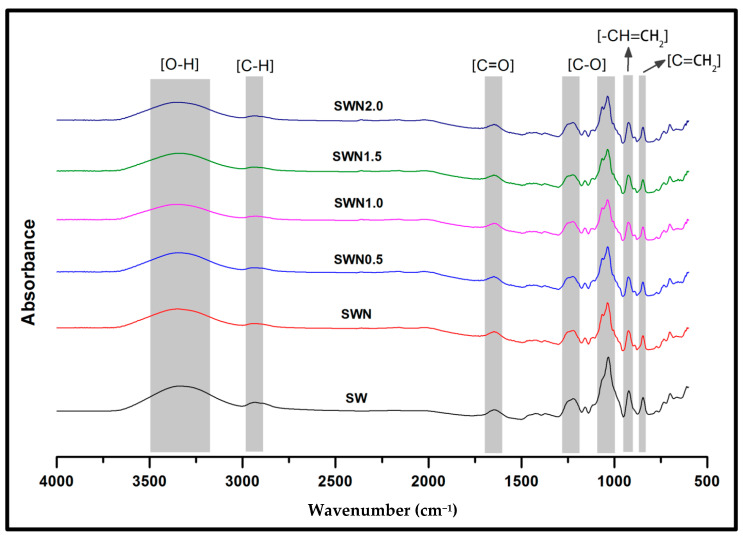
FT-IR spectra of seaweed, seaweed-neem, seaweed-neem, and gamma irradiated films.

The surface functional properties of seaweed (SW), seaweed–neem (SWN), and seaweed–neem gamma irradiated films (SWN0.5–2.0) are shown in Figure 2. The predominant functional groups were the –OH, C–H, C–O, and C=O. The broad peak observed from 3100 to 3500 cm^−1^ was assigned to the presence of the O–H groups, which decreased as the neem was incorporated into the seaweed film. The peak also decreased as the irradiated dosage of the films was increased compared to the control film. The absorbance from 3000 to 2900 cm^−1^ and peaks from 1500 to 1700 cm^−1^ were attributed to C–H and C=O stretching vibration, respectively, which indicated no significant difference relative to the control film. Similar results have been reported in previous studies [16]. The peaks at 1639.49 cm^−1^ were due to the carboxyl group (C=O) which are attributed to the stretching of carboxyl groups in the sulfated polysaccharides of seaweed. A strong band appeared at 1000 to 1220 cm^−1^ for the raw seaweed, which increased as irradiation dosage of the films increased. The assigned peak was attributed to the sulfate ester-stretching of the seaweed backbone [36]. Increased irradiation of the films resulted in the breaking down of polymeric chains into mono, di, and oligosaccharides. This was attributed to the increase in C–O stretching vibrations as irradiation dosage increased. A noticeable relatively broad peak was achieved after the film was irradiated and increased as radiation dosage increased, indicating enhanced photochemical reaction in the film attributed to increased molecular mobility and the effect of interfacial interaction of neem extract in seaweed matrix [37]. The strong peaks at 990 to 1050.85 cm^−1^ could be attributed to the polysaccharide glycosidic linkage stretching vibration suggesting that irradiation of the films did not alter the interfacial interaction between the neem extract in the seaweed matrix [38]. The peaks present between 1033 cm^−1^ is assigned to C–O and C=CH_2_ stretching vibrations of pyranose rings. These functional groups are very commonly present in all polysaccharides. The peaks at 923.90 cm^−1^ were due to the presence of 3,6-anhydro-d-galactose, and peaks at 844.82 cm^−1^ were assigned to d-galactose-4-sulphate [39]. This particular functional group in seaweed enables the ability of seaweed to form a helical secondary structure, which is important for gel and film formation. The presence of a weak bands at 740 cm^−1^ is assigned to the skeleton bending of pyranose ring in seaweed [40].

**Figure 3:**
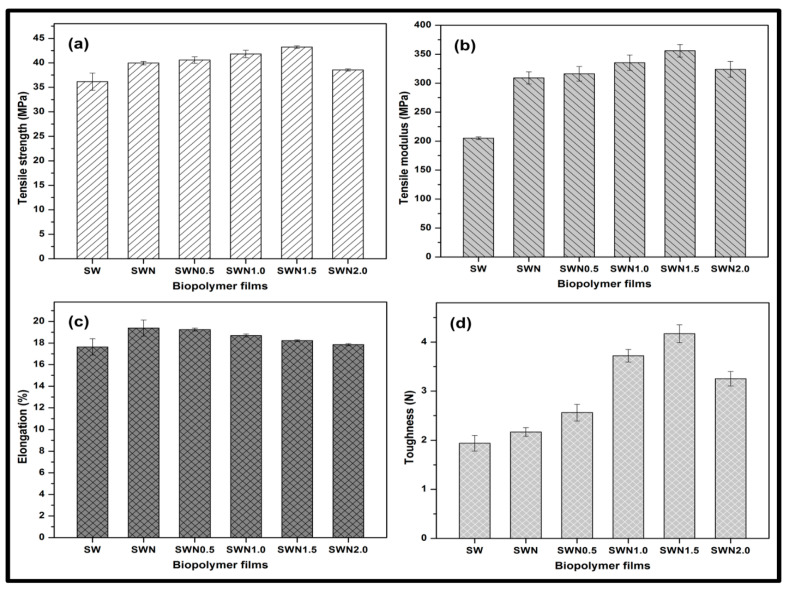
(**a**) Tensile strength and modulus (**b**) tensile modulus (**c**) elongation at break (**d**) toughness of seaweed, seaweed–neem, and seaweed–neem gamma irradiated film.

The tensile properties of seaweed, seaweed–neem, and seaweed–neem gamma irradiated film at 0.5, 1, 1.5, and 2 kGy are presented in Figure 3. The tensile strength of the control seaweed films, seaweed–neem film, and films under different radiation conditions are illustrated in Figure 3a. The tensile strength of the control seaweed was significantly lower compared to seaweed–neem films and the irradiated films suggesting the need for the reinforcement of the control film. The incorporation of neem leaves extract at 5% *w*/*w* % into the control seaweed films resulted in a moderate enhancement in tensile strength of the control seaweed films from 36.2 MPa to 40.0 MPa. The increase in the tensile strength observed in the seaweed–neem film indicated good dispersibility as a result of the interaction of the hydroxyl groups in the matrix [41,42]. However, a noticeable steep increase in the tensile strength was observed after the films were irradiated as the doses of the radiation of seaweed–neem films increased. A progressive increase occurred as irradiation increased until the seaweed–neem films were irradiated at doses of 1.5 kGy, after which a remarkable decrease in tensile strength occurred after the films were irradiated at 2 kGy. A similar trend was observed in Figure 3b. The tensile modulus (TM) of the biopolymer film exhibited a steady increase relative to the control seaweed film. The optimum TM was achieved at 1.5 kGy irradiated doses of the film at equivalent 350 MPa, beyond which there was a decrease in TM. Gamma radiation on the seaweed–neem composite film at doses of 1.5 kGy remarkably increased both the TS and TM of the film, indicating homogenous dispersion of the neem extracts in the seaweed matrix, which is attributed to the effective interfacial interaction. In addition, the effect of radiation of the films influenced the improved tensile properties of the biopolymer films. The enhancement in tensile properties of seaweed–neem film after gamma irradiation could be attributed to the enhanced hydrophobicity and the generation of free radicals, active sites within the composite seaweed film [43]. It could be inferred that the improved TS and TM of the biopolymer film depended on the interaction of the neem extracts in the matrix backbone of the film structure, even though, beyond 1.5 kGy, decreased TS and TM were achieved. This could be attributed to agglomeration of the component structure of the film when the radiation dose was increased to 2 kGy.

The elongation at break (EB) of the control seaweed film, seaweed–neem extract film, and the irradiated seaweed–neem films are represented in Figure 3c. The EB of the controlled film was observed to be 17.6%. However, the incorporation of 5% *w*/*w* neem leaves extract in the seaweed film increased the EB to 19.4%. The incorporation of the neem leaf extract could possibly increase the interfacial bonding interaction within the matrix [44]. The increased EB exhibited no significant difference with a 0.5 kGy dose of irradiation of the film. Although a very slight decrease in EB of the film between 1–2 Kgy doses of radiation suggested that there was no profound effect of radiation on the %EB of the film. In addition, the not very obvious difference in the percent EB of the seaweed neem films compared to the irradiated films at different doses further emphasized the significance of the interfacial interaction of the neem extract in the seaweed matrix [45]. Moreover, the interaction of the hydroxyl groups between seaweed and neem leaves and the extract resulted in improved compatibility and an increase in the EB of the film compared to the control seaweed [46]. The radiation dose treatment on the seaweed–neem film decreased %EB, and it was observed that the EB values of seaweed–neem film after radiation treatment were lower than the films EB values before irradiation [47], suggesting that the decreased EB values might be attributed to possible radiation-induced degradation of seaweed components when the irradiation doses increased. The tensile toughness as illustrated in Figure 3d exhibited a similar trend with the TS and TM of the films. The tensile toughness increased as the radiation dosage increased. The optimum tensile toughness was revealed at a 1.5 kGy radiation dosage, indicating that the mechanical properties of the incorporated films with neem extract were strongly enhanced by the effect of radiation dosage. 

**Figure 4:**
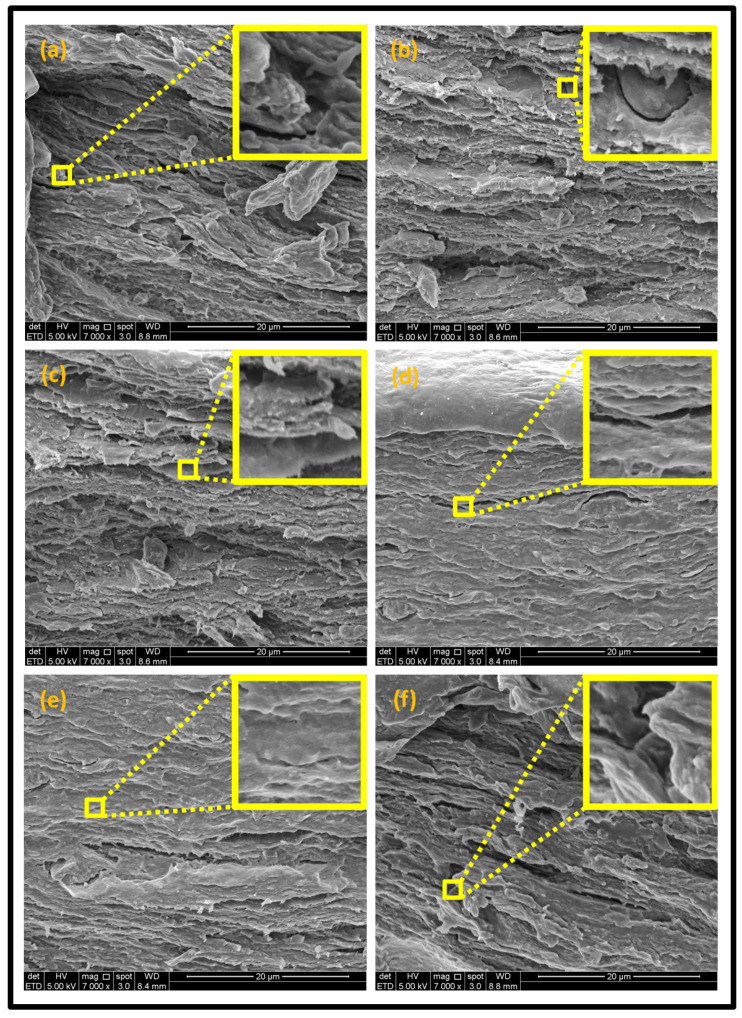
The fracture surface morphologies by Scanning Electron Microscope (SEM) of (**a**) seaweed film (control); (**b**) seaweed–neem film; (**c**) irradiated seaweed–neem at 0.5 kGy; (**d**) irradiated seaweed–neem at 1 kGy; (**e**) irradiated seaweed–neem at 1.5 kGy; (**f**) irradiated seaweed–neem at 2 kGy.

The SEM images were obtained to analyze the fracture morphology of the seaweed, seaweed–neem, and seaweed–neem gamma irradiated film as presented in Figure 4. The control seaweed-based film in Figure 4a exhibited a moderately fracture surface with noticeable cracks and pores due to the brittleness of seaweed matrix [48]. The predominance of cracks across the film surface indicated weak interfacial bonding, which could possibly create spaces for the diffusion of water molecules within the pores [49], suggesting lower resistance of the films to crack propagation across the boundaries. As a result, the weak interaction of the molecular chain decreased water resistance; in addition, reduced tensile properties of the films are more likely due to poor interfacial bonding as observed in Figure 3. The fracture surface of the non-irradiated seaweed–neem film is illustrated in Figure 4b. The fracture surfaces of the seaweed–neem films were rough, exhibited heterogeneous structure, and cavities at 5% *w*/*w* neem leaves extract concentration in which the voids were reduced compared to the control film. The fractured surface was rough with closed pores due to the incorporation of neem leaves extract in the seaweed matrix, which further improved the pore structure, morphology, and alignment of the fracture surface. The addition of neem extracts in the seaweed matrix improved the surface roughness compared to the control seaweed film, indicating clear changes in the morphological features due to the improved interfacial bonding as a result of the compatibility of the neem–seaweed matrix. This could be due to the formation of strong intermolecular interaction between seaweed and neem leave extract [50]. The significance of the interaction enhanced the compatibility of the seaweed–neem biopolymer films. A similar observation was reported by [51] on the fracture surface morphology of seaweed with oil palm shell and empty fruit bunch pulp fibers biocomposite films.

However, the SEM micrographs of the irradiated seaweed–neem film (Figure 4c) revealed a much improved surface morphology with clear evidence of smooth textural surface with a noticeable disappearance of pores. This could be attributed to the homogenous dispersion of the neem extracts in the seaweed matrix. In addition, the compatibility of the film components was strongly influenced by the effect of gamma radiation [52]. This was as a result of strong interaction of the seaweed and neem components in the composite seaweed–neem films. The strong interaction formed generally enhanced the tensile properties of the films after the radiation treatment. As the radiation doses increased Figure 4d,e, the cross section of the film revealed homogenous, smoother and dense fracture surface as the irradiation dosage of the films increased. The films’ structures were more homogenized and compacted, indicating that increased gamma irradiation dosage enhanced the structure of the film [16]. However, Figure 4f indicated a dense structure in the fracture surface with cracks noticed across the boundaries. This suggested weak interaction of the matrix structure irrespective of increased irradiation dosage. The observed surface could best be attributed to the effect of agglomeration. As a result, the film was susceptible to poor water resistance and possibly could affect the tensile properties of the film [53]. 

**Figure 5:**
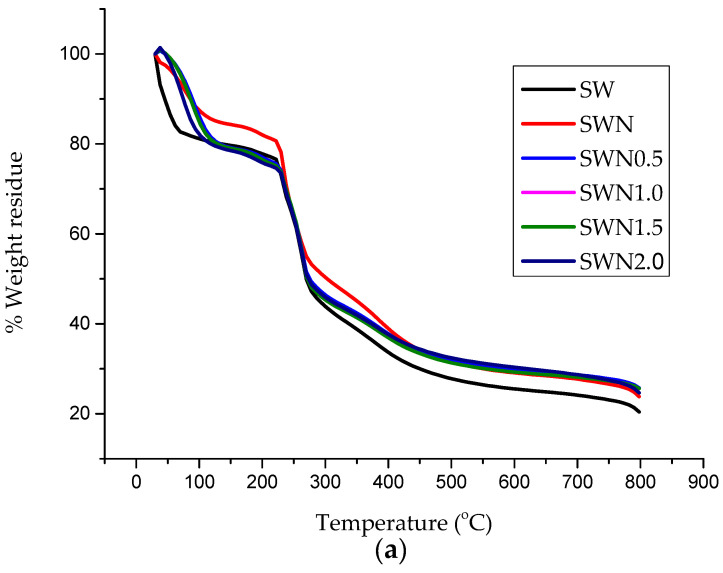

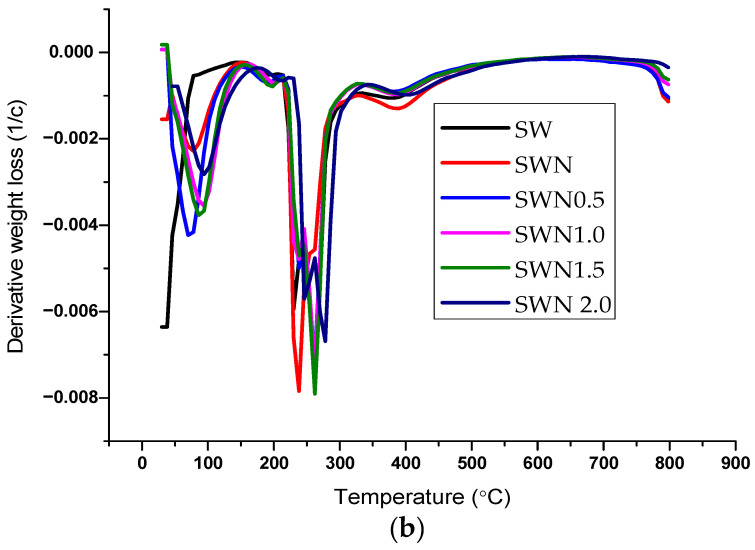
Thermal analysis indicating (**a**) TGA curve (**b**) DTG curve of the seaweed, seaweed-neem, and seaweed-neem irradiated films at different dosages.

Thermal properties of the seaweed based films were studied using Thermogravimetric analysis (TGA) to investigate the decomposition and thermal stability of the films (Figure 5). It was revealed that the initial temperature of degradation (T_on_), which is the point, the film began to disintegrate where deflection on the TGA curve was observed. The maximum temperature of degradation (T_max_) of the seaweed film was shifted to a higher temperature after the incorporation of neem leaves extract at 5 *w*/*w* % suggesting that the incorporation of neem leaves improved the thermal stability of the seaweed-based film [54]. This could be attributed to the strong intermolecular interactions of the film components as a result of higher energy required to break the intermolecular bonding, therefore leading to a higher T_on_ and T_max_. Moreover, the increase of thermal degradation temperature was also due to the presence of glycerol as a dispersing agent that could promote better seaweed–neem interactions [55]. The char residue content of irradiated seaweed–neem composite films was observed to increase after gamma irradiation. The gamma radiation caused chain scission or bond dissociations between seaweed and neem molecules due to the generation of free radicals. However, an increase in the percent residue confirmed the enhanced thermal stability of the irradiated seaweed–neem composite film [56] indicating the significance of irradiation of the films and the compatibility of the neem–seaweed intermolecular bonding interaction. The produced free radicals may react to change the structure of seaweed–neem film and strengthen the structure of the film. Hence, gamma radiation application could increase the thermal stability of the fabricated seaweed–neem composite film at 1.5 kGy. Gamma irradiation has the tendency to accumulate interfacial atoms and polymeric structural changes within the seaweed matrix [57]. The effect of irradiation possibly enhanced the thermal properties of the fabricated seaweed–neem composite films at the exposure of gamma radiation. Similarly, the seaweed–neem film exhibited higher percent residue compared to the control film, clearly indicating that the incorporation of the neem leaf extract in the seaweed matrix improved the thermal stability of the film. The (T_on_) and the (T_max_) was shifted to a higher temperature after the gamma radiation treatment on the seaweed–neem composite film optimally at 1.5 kGy at 273.5 and 262 °C. Further increase in radiation treatment by 2.0 kGy resulted in a decrease in the degradation temperature and percent residue of the film by 251.21 °C and 30.2%, respectively. These findings were in good agreement with a previously reported study by [58], and it was revealed that the crosslinking of chitosan nanocomposite film with citric acid and magnesium oxide demonstrated an increase in thermal stability to up to 1.5 kGy of a gamma radiation dose. Generally, the TGA results of the irradiated seaweed–neem composite films indicated that the developed films exhibited higher thermal stability. 

**Figure 6:**
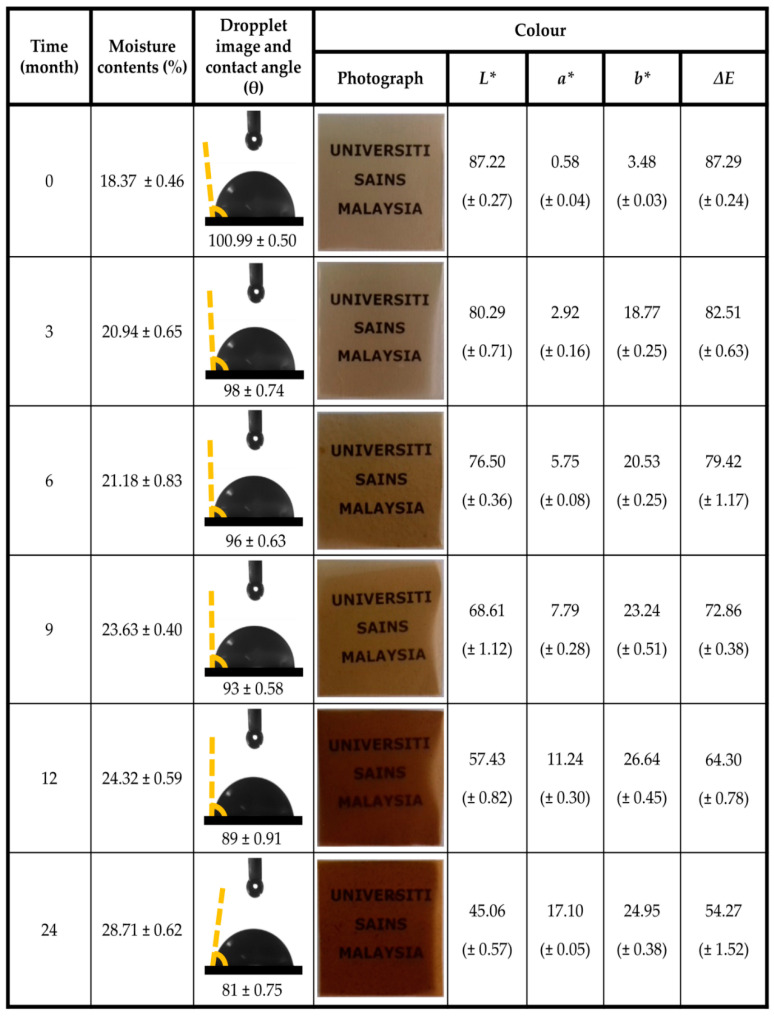
Moisture content, contact angle, and color properties of seaweed–neem films at different doses of radiation.

The percent moisture content of the control film and irradiated film measured at room temperature and 50% RH is presented in Figure 6. The irradiated film used for the analysis was obtained from the optimum irradiation dosage of 1.5 kGy from the preliminary study. It can be observed that a steep steady increase of the moisture content of the film was achieved compared to the control film. However, it can be inferred that the slight steady increase of moisture content relative to the storage period had no negative effect on the properties of the film [59]. This is indicated from the range of the average moisture content value achieved for the control film compared to the irradiated film at the end of 24 months of storage. This is in conformity with a decrease in the hydrophobicity exhibited by the irradiated film over a prolonged storage period. It is revealed that the irradiated film retained its hydrophobic properties until nine months of storage, suggesting interplays within the matrix structure resulted in enhanced compatibility of the film and moisture resistance [60]. Beyond this storage period, a steady decrease in the hydrophobic behavior of the film was noticed. The color of the film determines appearance, which influences consumer acceptance [54]. Observation showed that, as the storage period increased, the film color moderately decreased, although the effect of the color change had no significant influence on the stability of the film throughout the storage period. Similarly, there was no significant difference in the color of the film until nine months of storage period compared to the control film, which implies that the irradiated film properties were not affected during the storage period. As observed in the color parameters, the films exhibited good transparency as indicated by the values of lightness (L*). The increase in storage time resulted in a decrease in the film’s transparency as indicated in the value of lightness. However, the increase in the storage time resulted in the increased value of greenness (a*). The color parameter illustrating the color of the films at storage is indicated by the (b*) value. It was revealed that the value of b* of the films increased as the storage time increased. 

The effect of storage time on the properties of the ISN films as packaging material were evaluated at ambient conditions (25 °C with 50% RH). The effect of storage time on the tensile properties of the packaging film such as tensile strength (TS), tensile modulus (TM), and elongation at break (EAB) were shown in Table 1.

Findings indicated that the TS and TM decreased as the storage time increased. As compared to the control film, the storage period of 24 months exhibited the lowest TS and TM. Conversely, the EB increased as the storage time increased. The highest EB was achieved at 24 months of storage. However, a steep decrease in TS and TM of the developed ISN film as storage time increased demonstrated that the storage stability of the irradiated films within 24 months was not significantly affected by the storage period, even though a slight steady decrease in the tensile films’ tensile strength was achieved as EAB increased [61]. The TS and TM values demonstrated a relatively steep decrease as the storage period increased. The observed moderately decrease in the value of the TS and TM of the ISN films could be attributed to the decrease in the compatibility of the intermolecular bonding of the components in the matrix during the storage period. The tensile behavior of the films were closely related to its microstructure [62]. This is reflected in the microstructural changes observed during storage. The developed film presented a gradual increase in EAB value as the storage time increased [60]. The increase in EAB value signifies less intermolecular interaction of the component of the film [63]. After the storage period, the film increased in rigidity, becoming more resistant and brittle, which may be due to the greater compactness of the matrix associated with moisture loss as observed in Table 1 [64]. The TS and TM values of the ISM films were compared to those of commercial high density polyethylene (HDPE) and low density polyethylene (LDPE) under the investigated operational condition [65,66]. The tensile properties and storage stability of the currently developed ISN film proved that the film can be potentially used as packaging material.

**Figure 7:**
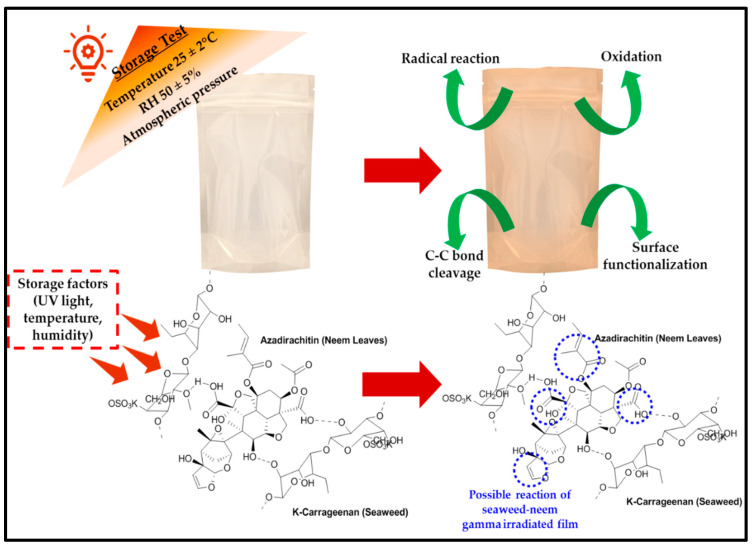
Schematic representing possible mechanistic action of the irradiated biofilms for active packaging.

The application of biopolymer films has proven to be a suitable alternative to conventional plastics for active packaging. However, in this study, the possible mechanistic action of the irradiated antimicrobial films is illustrated in Figure 7. The capacity of the films to retain its hydrophobic properties until after nine months of storage revealed that an irradiated surface of the films provided active sites for better interactions with enhanced surface functional properties.

**Figure 8:**
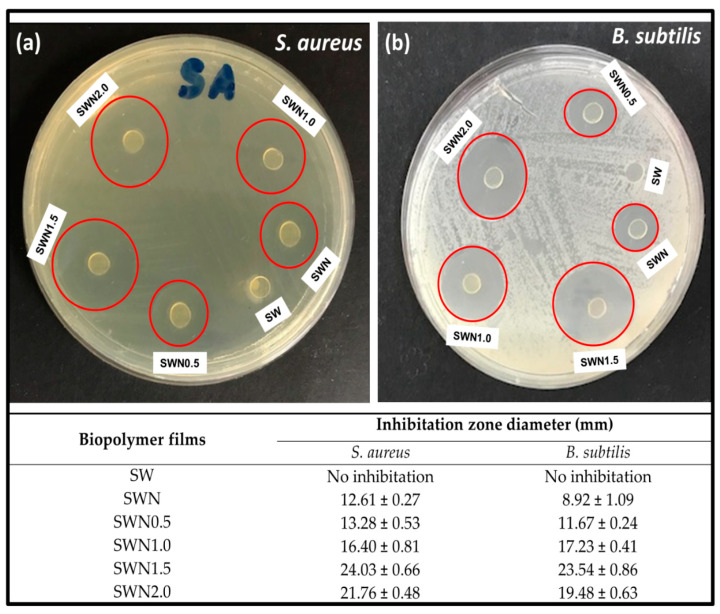
Photographic images of inhibition zone measurement results of seaweed film (SW), seaweed–neem composite film (SWN) and irradiated seaweed–neem film (SWN) at different dosage (kGy) against; (**a**) *Staphylococcus aureus*; (**b**) *Bacillus subtilis*.

The antimicrobial activities of the films were measured using the agar diffusion method with film in a disc shape. The diameter of inhibitory zones surrounding the film discs was used to evaluate the inhibitory and antimicrobial activities of the irradiated films [67]. The antimicrobial activities of the seaweed-based films tested against *Staphylococcus aureus*, and *Bacillus subtilis* is indicated in Figure 8. As expected, the seaweed film (control) exhibited no inhibitory zones against the tested bacteria. However, the incorporation of 5% *w*/*w* neem leaves extract into the control film demonstrated inhibitory activities against *Staphylococcus aureus* and *Bacillus subtilis* with the diameter of inhibition zone of 12.61 ± 0.27 mm and 8.92 ± 1.09 mm, respectively [68], suggesting that the difference in the antimicrobial activities against bacteria was attributed to the lipid bilayer composition of bacterial strains and the degree of depolarization and permeability of the cell walls [69]. In addition, the antimicrobial activity of neem leaves extract-based seaweed films might be due to the presence of effective components such as carotenoids, phenolic compounds, flavonoids, triterpenoids, ketones, valavinoids, saponins, gilcosides, steroids, and tetra-triterpenoids azadirachtin in the neem leaves [21]. This could be attributed to the essential components of incorporated neem extracts in the seaweed matrix, which served as the principal antibiotics that could serve as a defensive mechanism against different pathogens [70]. 

However, the irradiation of the seaweed–neem films at different dosages exhibited increased inhibitory zones against *Staphylococcus aureus* and *Bacillus subtilis* until a radiation dosage of 1.5 kGy was applied. It can be inferred that an increase in irradiation dosage significantly enhanced the antimicrobial activities of the films against *Staphylococcus aureus* compared to *Bacillus subtilis.* Similarly, the irradiation of the films resulted in the induced free radicals in the film as a result of increased bonding interactions between neem extracts in the seaweed matrix in the film structure, thereby enhancing the antimicrobial activity of the film [71].

Although the films exhibited a less inhibitory effect against the investigated bacteria after an irradiation dosage of 2 kGy was applied compared to the effect of other irradiation dosage without a significant negative effect. These suggested that the interfacial interaction of the neem extracts in the seaweed matrix and subsequently the effect of irradiation of the films enhanced antimicrobial activities against *Staphylococcus aureus* and *Bacillus subtilis.* This result was in good agreement with a previous study on polyamide coated low density polyethylene (LDPE) film with active compounds (sorbic acid, carvacrol, thymol, and rosemary oleoresin), where the antimicrobial activity of the films was found to get retained when exposed to 1–3 kGy [72].

**Figure 9:**
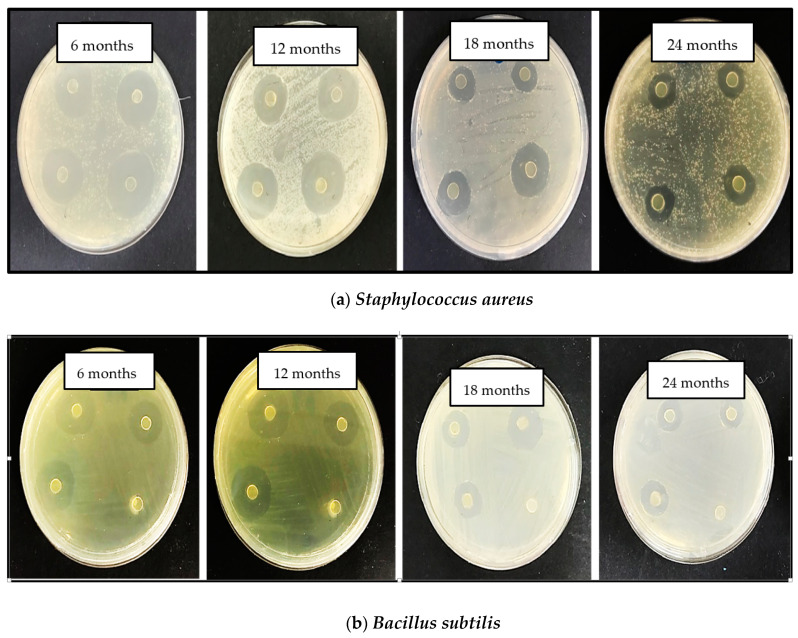
Photographic images of inhibition zone measurement results of seaweed–neem film irradiated at optimized dosage of 1.5 kGy against; (**a**) *Staphylococcus aureus*; (**b**) *Bacillus subtilis*.

The stability of ISN film at storage conditions of 25 °C with 50% RH against *Staphylococcus aureus* (*S. aureus*) and *Bacillus subtilis* (*B. subtilis*) was investigated for 24 months using optimal irradiation dosage of 1.5 kGy as shown in Figure 9. Six months of storage were revealed, and the inhibition zone of ISN film was 13.15 mm against *S. aureus* and 22.05 mm against *B. subtilis*. The extent of inhibition of microbial activities slightly decreased after 24 months of storage compared to the effect of microbial activities before storage. The values of the inhibition zone achieved for *S. aureus* and *B. subtilis* were 10.03 mm and 18.78 mm, respectively. It was clearly evident that a slight decrease in the inhibition zone at 24 months of storage compared to six months of storage indicated that the ISN film exhibited stability against microbial activities during storage despite the prolonged storage period, suggesting that the antimicrobial effect of the film could be attributed to the relatively high concentration of ethyl. Alpha-d-glucopyranoside, ethyl 9, 12, 15-octadecatrienoate, and phytol compounds [66], which were the predominant compounds in the neem plant extract. The enhancement of the antimicrobial activity after a prolonged storage period could also be attributed to the significance of low constant storage temperature (25 °C), which optimally maintained the major active compounds of neem leaf extract such as nimbolide, phytol, and phenolic acid from evaporating. The authors in [73] reported that low storage temperature has a high tendency to maintain major effective compounds from evaporating in films compared to high storage temperature. The results of the effect of storage on antimicrobial activities of ISN films obtained in this study are in agreement with previous studies [74], which implies that, under the low constant temperature, ISN biodegradable packaging film exhibited effective antimicrobial activities against *S. aureus* and *B. subtilis* over a prolonged storage period as evidenced in enhanced antimicrobial activities even after a 24-month storage period. 

## 4. Conclusions

This study successfully fabricated an antimicrobial seaweed–neem extract irradiated film. The films were characterized and storage functional properties of the films were investigated for sustainable packaging application. The seaweed-based films were incorporated with 5% *w*/*w* neem leaves extract and were treated with an optimized radiation dosage of 1.5 kGy gamma radiation. The irradiated films exhibited enhanced tensile properties and thermal stability of the film with excellent antimicrobial activity towards *Staphylococcus aureus* and *Bacillus subtilis*, which is significant for effective storage for packaging. The irradiated films demonstrated reduced water permeability, suggesting improved hydrophobic properties. The morphology of the irradiated composite film indicated a well aligned smoother surface compared to the control seaweed–neem film. This can be attributed to the improved tensile properties of the irradiated films as a result of good intermolecular interaction of neem extracts in the seaweed matrix. During the storage period, the film exhibited an optimum storage stability and indicated no significant changes in the physical appearance. The film revealed optimum enhanced tensile properties, which is an indication of improved mechanical properties exhibited by the film. The inhibition of microbial activities slightly decreased after irradiation of 2 kGy, compared to the effect of microbial activities of the control film, and the decrease did not affect the antimicrobial activities of the film. Accordingly, the stability of the irradiated film during prolonged storage period indicated the suitability of film for sustainable food packaging with enhanced antimicrobial activities. 

## Figures and Tables

**Table 1:** Effect of storage period on tensile properties of the packaging film.

Storage Period	TS	TM	EAB
6 months	43.31 ± 0.21	350.27 ± 0.12	19.22 ± 0.08
12 months	42.24 ± 0.15	321.56 ± 1.14	19.55 ± 0.07
18 months	41.89 ± 0.02	310.60 ± 1.64	19.98 ± 0.11
24 months	38.31 ± 0.21	305.19 ± 2.33	20.38 ± 0.09

## Data Availability

Not applicable.
